# Prevalence of Physical Inactivity and Sedentary Behavior Among Adults in Armenia

**DOI:** 10.3389/fpubh.2020.00157

**Published:** 2020-05-05

**Authors:** Antonina Tcymbal, Diana Andreasyan, Stephen Whiting, Bente Mikkelsen, Ivo Rakovac, João Breda

**Affiliations:** ^1^Department of Sport Science and Sport, Friedrich-Alexander University of Erlangen-Nuremberg, Erlangen, Germany; ^2^National Institute of Health, National Health Information Analytic Center, Yerevan, Armenia; ^3^WHO European Office for the Prevention and Control of Noncommunicable Diseases, Division of Noncommunicable Diseases and Promoting Health Through the Life-Course, WHO Regional Office for Europe, Moscow, Russia; ^4^EPIUnit—Instituto de Saúde Pública, Universidade de Porto, de Porto, Portugal; ^5^Division of Noncommunicable Diseases and Promoting Health Through the Life-Course, WHO Regional Office for Europe, Copenhagen, Denmark

**Keywords:** physical inactivity, sedentary behavior, physical activity levels, prevalence, GPAQ, adults

## Abstract

Physical inactivity and sedentary behavior are risk factors for developing non-communicable diseases. This study analyzed current levels of physical inactivity and sedentary behaviors among the adult population of Armenia. Data were collected through a national STEPS survey of non-communicable diseases risk factors on a nationally-representative sample of 2,380 participants aged 18–69 years in Armenia in 2016. The Global Physical Activity Questionnaire was used to assess physical activity levels. Two out of ten people (21.6%) in Armenia did not meet the minimum levels of physical activity recommended by WHO to protect health. 13.2% of population spent over 8 h per day sitting, 47.2% were inactive at work and 32.4% did not do any transport-related physical activity. Only 13.8% of participants were physically active during leisure time. Specific groups with relatively high levels of physical inactivity were older adults, residents of Yerevan, people with lower levels of education, the unemployed and people who were retired. Sedentary behavior was more common among men, students, people who were retired, unemployed, residents of Yerevan, and adults aged under 30 and over 45 years.

## Introduction

It is clear that physical activity (PA) is essential for the maintenance of physical and mental health (1–3). Physical inactivity can be defined as not achieving the minimum levels of physical activity, as recommended by the World Health Organization (WHO) (2). Investing in policies that encourage physical activity can directly contribute to the achievement of the 2030 Sustainable Development Goals, and the WHO's Global Action Plan for Physical Activity 2018–2030 (4) as well as the European Physical Activity Strategy 2016–2025 (3) and encourage countries to strengthen monitoring and surveillance of physical activity to help guide national policy action.

Physical inactivity is a key risk factor for the development of many non-communicable diseases (NCD) such as heart disease, stroke, type 2 diabetes and breast and colon cancer (1, 4). PA can also be beneficial to mental health and increase social inclusion (5, 6). While meeting the WHO recommended levels of physical activity can protect health (2), there are indications that sedentary behavior is also an independent risk factor for many NCDs (7–11). Sedentary behavior (SB) can be defined as “any waking behavior characterized by an energy expenditure ≤ 1.5 METs while in a sitting or reclining posture” (12). It is important to be clear that SB is not a synonym for physical inactivity, rather, they are both separate and independent risk factors for chronic diseases (9, 13).

In Armenia, the prevalence of NCD-related mortality has been increasing (14). The prevalence of risk factors for NCDs such as unhealthy diet and physical inactivity is high and around half of adults (51.2%) are overweight or obese (15). Consequently, the prevention and control of NCDs are a priority of the Armenian Government. In order to address this issue, quality data related to risk factors such as physical inactivity and sedentary behavior are essential to enable the development of effective and targeted policies, strategies, and interventions to promote health and prevent NCDs.

The WHO STEPwise approach to surveillance (STEPS) survey of NCD risk factors was developed to collect high quality, nationally representative data which can be used to help guide policy development for the prevention and control of NCDs. It allows assessment of the prevalence of behavioral risk factors, such as physical inactivity and SB, as well as biological risk factors, such as raised blood pressure blood cholesterol, and glucose levels (16). Analysis of data collected through the STEPS survey enables estimation of population PA levels and prevalence of SB for different socio-demographic groups at the country level.

In 2016, Armenia implemented the STEPS survey for the first time. Based on the data collected, the current study aimed to provide the first national estimate of PA levels and sedentary behaviors for the Armenian population disaggregated by socio-demographic factors such as gender, age, location of residence, level of education and occupation.

## Methods

### Study Design and Sampling

Data were collected through the national STEPS survey of NCDs risk factors conducted in 2016. The survey was implemented according to the WHO STEPS methodology (17). It involved interviews of participants to assess behavioral risk factors and health history related to NCDs (Step 1), physical/anthropometric measurements (Step 2) and biochemical measurements (Step 3). The sample was defined using a multi-stage cluster sampling method based on demographic data regarding the adult population of Armenia (14). The STEPS survey methodology, translated questionnaire, informed consent of participation and informative letter were approved by the Ethics Committee of the National AIDS Prevention Center of Armenia and the WHO (14). The measurements were obtained during face-to-face interviews. Data collectors were previously trained by WHO experts (14).

### Data Collection Instrument

The STEPS instrument includes the Global Physical Activity Questionnaire (GPAQ) which has been validated and assessed to be suitable and acceptable for monitoring physical activity at the population level (18). The GPAQ allows assessment of the frequency and duration of PA in three domains: at work, for transportation and for leisure or recreational activities. It also differentiates between moderate- and vigorous-intensity PA for the work and recreational PA domains (19). This enables calculation of the time spent on PA in each of the domains separately as well as total amount of PA, reported in minutes per week or in metabolic equivalent of task (MET). One MET is equivalent to a caloric consumption of 1 kcal/kg/hour which is the energy cost of sitting quietly (20). According to the GPAQ analysis guide, 4.0 MET equates to a moderate MET minute value for work-related and recreational PA, 8.0 MET equates to a vigorous-intensity value for work-related and recreational PA, and 4.0 MET equates to transportation-related PA (20).

The information collected through the GPAQ allows estimation of the proportion of the population that are meeting the WHO global recommendations on PA for health. According to the recommendations, adults aged over 18 years should either achieve a minimum of 150 min of moderate-intensity physical activity, or 75 min of vigorous-intensity physical activity, or an equivalent combination of moderate- and vigorous-intensity physical activity that is the equivalent of at least 600 MET-minutes per week. It is also recommended to include muscle-strengthening activities involving major muscle groups on at least 2 days per week (2). The GPAQ has an additional item for recording time spent in sedentary activities and collects information on the socio-demographic characteristics of participants including sex, age, occupational status, educational levels, and location of residence (19).

### Data Collection Process

The STEPS survey in Armenia was conducted among the 18–69 years old population from 13 September through 25 December 2016. The interviewers visited over 5 600 households and completed 2 380 interviews. The response rate was 42%. Main reasons for the somewhat low response rate were refusals to participate in the interviews (especially in the capital), and to much smaller extent errors in the addresses recorded in the unified population register.

From the total sample, 31 participants did not provide socio-demographic information and 100 participants provided incomplete or implausible responses to PA questions (e.g., more than 16 h of physical activity per day) and were excluded from the final dataset.

### Data Analysis

For comparative purposes, the sample was divided into four age groups: 18–29, 30–44, 45–59, and 60–69. Educational levels were recoded into three levels: (1) secondary school (or lower) completed; (2) high school completed; (3) college/university (or higher degree) completed. The proportion of the population in Armenia was 36.5% rural and 63.5% urban (14). 35.6% of the population (more than half of all urban residents) lived in the capital city, Yerevan, which makes it unique from the perspective of access to a PA promotive environment, despite being in cities with more sport facilities, studies have shown that inhabitants of urban sprawls have higher level of physical inactivity (21, 22). Therefore, the sample was divided into three groups according to location of residence: rural, urban and Yerevan residents. The sample was also divided into five groups by occupation: “Employed,” “Student,” “Homemaker,” “Retired,” and “Unemployed.”

SB was defined as sitting for over 8 h daily as studies have indicated that this amount of SB can be harmful to health and increase the risk of NCDs and all-cause mortality (7, 23, 24).

Pearson's Chi-square test was used for comparison of proportions and Wilcoxson rank test or Kruskal-Wallis test used for comparison of continuous variables such as average amount of PA and sedentary time. Data were weighted to account for cluster design, age, sex, and non-response by age and gender. Statistical analysis was conducted in R version 3.4.3 using the Survey package (25).

## Results

The study sample consisted of 2,249 (52% male) subjects aged 18–69 years. Socio-demographic characteristics of population are presented in Table 1.

**Table 1:** Socio-demographic characteristics of the study population.

	**Percentage of population (weighted), %**
Gender	
Men	52
Women	48
Age group	
18–29	34.3
30–44	29.3
45–59	26.6
60–69	9.8
Education	
Secondary school (or lower)	48.2
High school	26.1
College/University (or higher degree)	25.7
Residence	
Yerevan	41.5
Urban	23.5
Rural	34.0
Occupation	
Employed	38.7
Student	7.0
Homemaker	25.0
Retired	5.1
Unemployed	22.8

### General Description of Physical Activity Level and Sedentary Behavior in Armenia

21.6% (95% CI: 18.7–24.4) of adults did not meet the WHO PA recommendations of at least 600 MET-minutes per week. Almost half of participants (47.2%, 95% CI: 43.1–51.2) were inactive at work and around one third (32.4%, 95% CI: 28.2–36.5) were not active for transport purposes (e.g., walking or cycling). Less than 15% of participants were physically active in leisure time. 78.9% (95% CI: 76.4–81.4) of the population did not engage in any vigorous-intensity activity. The average amount of total PA time per day was 209.0 min (95% CI: 193.4–224.5), and the median PA time per day was 120 (IQR: 30–345) minutes. The mean PA in MET-minutes per week for the adult population of Armenia was 6932.5 (95% CI: 6381.6–7483.5) and the median was 3520 (IQR: 840–10846) MET-minutes per week. Mean sitting time per day was 228.6 minutes (95% CI: 213.8–243.4) and the median was 180 (IQR: 120–330) minutes. 13.2% (95% CI: 10.7–15.7) of the population spent more than 8 h per day sitting.

### Socio-Demographic Factors

#### Gender

In general, men were more physically active (see Table 2). They did significantly more physical activity than women when calculated in MET-minutes per week (*p* = 0.02). 30.8% of men engaged in vigorous-intensity PA which was three times higher than women (*p* < 0.001). The only indicator for which women were more active than men was for transport-related PA (*p* = 0.03). Conversely, men overall spent more minutes sitting per day (*p* = 0.004). 15.6% of men spent more than 8 h per day sitting, compared to 10.6 % of women, *p* = 0.009.

**Table 2:** Indicators of physical activity and sedentary behavior for gender.

	**Gender**		
	**Men**	**Women**	***p*-value**
Meet WHO recommendations on PA, % (95% CI)	77.5 (73.4–81.6)	79.4 (76.3–82.5)	*p* = 0.42
No work related PA, % (95% CI)	46.5 (40.6–52.5)	47.9 (43.7–52.1)	*p* = 0.67
No transport PA, % (95% CI)	35.4 (29.3–41.6)	29.1 (25.5–32.6)	*p* = 0.03
No recreation related PA, % (95% CI)	85.4 (81.8–88.9)	87.1 (84.7–89.5)	*p* = 0.41
No vigorous PA, % (95% CI)	69.2 (65.0–73.3)	89.5 (87.5–91.5)	*p* < 0.001
Sedentary behavior, minutes (95% CI)	248 (224–271)	208 (195–221)	*p* = 0.004
Sitting more than 8 hours per day, % (95% CI)	15.6 (12.0–19.3)	10.6 (8.0–13.2)	*p* = 0.009
Average amount of PA in minutes per day, mean (95% CI)	222 (198–247)	194 (180–208)	*p* = 0.25
Average amount of PA in MET/min/week, mean (95% CI)	7,983 (7,097–8,870)	5,791 (5,372–6,210)	*p* = 0.02

#### Age

The analysis showed that there are statistically significant differences between age groups for all indicators of physical activity and sedentary behavior except proportion of people sitting more than 8 h per day (see Table 3).

**Table 3:** Indicators of physical activity and sedentary behavior for age.

	**Age**				
	**18–29**	**30–44**	**45–59**	**60–69**	***p*-value**
Meet WHO recommendations on PA, % (95% CI)	81.1 (76.9–85.4)	80.7 (76.8–84.7)	74.1 (69.1–79.2)	73.6 (68.4–78.8)	*p* = 0.013
No work related PA, % (95% CI)	51.0 (45.2–56.7)	40.7 (35.3–46.2)	45.7 (40.2–51.3)	57.1 (51.2–63.0)	*p* < 0.001
No transport PA, % (95% CI)	26.4 (20.8–32.0)	32.5 (26.4–38.7)	39.1 (33.6–44.6)	34.7 (29.1–40.2)	*p* < 0.001
No recreation related PA, % (95% CI)	80.9 (76.7–85.1)	85.5 (81.2–89.9)	91.6 (89.3–93.9)	92.1 (89.4–94.9)	*p* < 0.001
No vigorous PA, % (95% CI)	81.0 (76.4–85.5)	72.6 (68.0–77.2)	79.6 (75.6–83.6)	88.8 (85.2–92.5)	*p* < 0.001
Sedentary behavior, minutes (95% CI)	233.5 (212–255)	209.6 (188–231)	232.7 (213–252)	257.2 (235–279)	*p* = 0.002
Sitting more than 8 h per day, % (95% CI)	13.5 (9.6–17.4)	11.4 (7.6–15.2)	13.9 (10.2–17.7)	15.9 (11.2–20.6)	*p* = 0.49
Average amount of PA in minutes per day, mean (95% CI)	184 (160–208)	249.3 (224–275)	209.2 (186–232)	174.6 (148–202)	*p* = 0.001
Average amount of PA in MET/min/week, mean (95% CI)	5,830 (5,010–6,650)	8,532 (7598–9465)	7,141 (6,293–7,988)	5,434 (4,539–6,331)	*p* < 0.001

Young people aged 18–29 years had low levels of PA. While only 18.9% did not meet WHO recommendations, in terms of total amount of PA and SB they were closer to the oldest age group than to the 30–44 year old group.

The most active age group were people between 30 and 44 years old. They were significantly more engaged in work-related and vigorous-intensity PA, achieved more PA per day (249.3 min), per week (8532 MET-min), and spent less time sitting per day (209.6 min).

People aged 60–69 years were the least active age group. 57.1% did no work-related PA and only 7.9% were active for recreation or leisure. The average amount of time spent sitting per day was 257.2 min and average amount of PA was 174.6 min per day. 26.4% did not meet WHO recommendations on PA.

#### Residence

There are statistically significant differences between rural, urban, and Yerevan residents for all indicators of PA except time spent on sedentary activities per day (see Table 4). Yerevan residents showed the lowest levels of PA between the three population groups: 56.7% did not do any work-related PA, 19.4% spent more than 8 h per day sitting, 28% did not meet WHO recommendations. People from rural areas were the most active. Only 14.8% of them did not meet WHO recommendations, less than 8% spent more than 8 h per day sitting, and the average amount of total PA was 260.9 min per day. However, they mostly did work- and transport-related physical activity; only 8.9% did PA for recreation or leisure which was half of the level of Yerevan residents (18.1%).

**Table 4:** Indicators of physical activity and sedentary behavior for residence.

	**Residence**			
	**Yerevan**	**Urban**	**Rural**	***p*-value**
Meet WHO recommendations on PA, % (95% CI)	72.0 (66.0–78.0)	79.7 (75.5–84.0)	85.2 (82.2–88.1)	*p* < 0.001
No work related PA, % (95% CI)	56.7 (48.2–65.2)	48.9 (43.0–54.8)	34.6 (29.4–40.0)	*p* = 0.024
No transport PA, % (95% CI)	37.1 (28.9–45.3)	34.4 (28.9–40.0)	25.4 (20.6–30.2)	*p* < 0.001
No recreation related PA, % (95% CI)	81.9 (77.2–86.5)	86.6 (83.0–90.1)	91.1 (88.3–93.9)	*p* = 0.001
No vigorous PA, % (95% CI)	83.0 (79.0–87.0)	85.3 (81.4–89.2)	69.8 (65.4–74.1)	*p* = 0.001
Sedentary behavior, minutes (95% CI)	248 (215–281)	216 (196–236)	213 (200–227)	*p* = 0.52
Sitting more than 8 h per day, % (95% CI)	19.4 (13.9–25.0)	10.9 (7.5–14.3)	7.4 (4.8–9.9)	*p* < 0.001
Average amount of PA in minutes per day, mean (95% CI)	170.9 (139–203)	198.8 (176–222)	260.9 (238–284)	*p* < 0.001
Average amount of PA in MET/min/week, mean (95% CI)	5,676 (4,552–6,800)	6,325 (5,482–7,167)	8,832 (7,997–9,667)	*p* < 0.001

#### Education

People with a higher level of education were more engaged in leisure-time PA and more likely to meet WHO recommendations on PA (see Table 5). People with lower levels of education were more engaged in vigorous-intensity PA and achieved a higher average amount of PA. The proportion of people sitting more than 8 h per day was significantly higher among people with a college/university or higher degree (17.8%).

**Table 5:** Indicators of physical activity and sedentary behavior for education groups.

	**Education level**			
	**Secondary school**	**High school**	**College/University**	***p*-value**
Meet WHO recommendations on PA, % (95% CI)	75.2 (70.9–79.5)	81.6 (77.6–85.6)	81.2 (76.6–85.2)	*p* = 0.03
No work related PA, % (95% CI)	45.5 (40.4–50.6)	45.2 (39.2–51.2)	52.3 (45.3–59.2)	*p* = 0.15
No transport PA, % (95% CI)	35.7 (30.7–40-8)	29.7 (24.7–34.7)	28.7 (22.2–35.2)	*p* = 0.03
No recreation related PA, % (95% CI)	88.9 (86.2–91.6)	89.3 (86.5–92.2)	77.9 (72.9–82.9)	*p* < 0.001
No vigorous PA, % (95% CI)	76.0 (72.3–79.8)	78.7 (74.0–83.4)	84.6 (80.4–88.7)	*p* = 0.018
Sedentary behavior, minutes (95% CI)	225.2 (207–243)	226.3 (210–243)	237.2 (208–266)	*p* = 0.9
Sitting more than 8 h per day, % (95% CI)	11.7 (8.6–14.8)	11.5 (8.5–14.6)	17.8 (12.6–23.1)	*p* = 0.02
Average amount of PA in minutes per day, mean (95% CI)	219.8 (198–241)	227.0 (200–254)	170.3 (148–193)	*p* = 0.027
Average amount of PA in MET/min/week, mean (95% CI)	7,447 (6,698–8,196)	7,614 (6,567–8,660)	5,277 (4,522–6,033)	*p* = 0.012

#### Occupation

There are statistically significant differences between occupational groups for all physical activity and sedentary behavior indicators (see Table 6). The most inactive group was “unemployed”; 33.6 % did not meet WHO recommendations on PA. Other groups which were also in the low-level PA category were “retired” people, which consisted mostly of older adults who were less physically active. This group had very low engagement in recreation PA−4.9%. “Employed” and “homemaker” groups were more engaged in work-related physical activity. Homemakers had the lowest levels of sedentary behavior with only 5.3% sitting more than 8 h per day and an average sitting time of 179.6 min per day. Among students, 86.1% met WHO recommendations on PA, although this group had the lowest average amount of PA (4009 MET-minutes per week).

**Table 6:** Indicators of physical activity and sedentary behavior for different occupation groups.

	**Employed**	**Student**	**Homemaker**	**Retired**	**Unemployed**	***p*-value**
Meet WHO recommendations on PA, % (95% CI)	81.6 (77.2–85.9)	86.1 (78.6–93.7)	83.8 (79.9–87.6)	68.5 (60.9–76.0)	66.4 (60.2–72.5)	*p* < 0.001
No work related PA, % (95% CI)	42.3 (36.0–48.5)	69 (56.2–81.7)	38.6 (33.1–44.0)	55 (46.2–63.9)	58.7 (52.2–65.2)	*p* = 0.67
No transport PA, % (95% CI)	35.2 (28.4–42.0)	19.1 (10.2–28-0)	29.2 (24.0–34.3)	38.9 (31.8–46.0)	32.6 (26.8–38.4)	*p* = 0.03
No recreation related PA, % (95% CI)	82.8 (78.6–87.1)	69.6 (58.4–80.8)	88.1 (85.1–91.2)	95.1 (92.6–97.5)	93.2 (90.6–96.0)	*p* = 0.001
No vigorous PA, % (95% CI)	75.5 (71.5–79.6)	74.3 (63.1–85.5)	80.1 (75.3–85.0)	92.3 (87.2–97.4)	83.1 (78.0–88.1)	*p* = 0.014
Sedentary behavior, minutes (95% CI)	227.5 (202–253)	255.2 (204–307)	179.2 (165–194)	284.9 (250–320)	268.4 (244–292)	*p* < 0.001
Sitting more than 8 hours per day, % (95% CI)	13.7 (9.7–17.7)	18.4 (7.8–29.0)	5.3 (2.9–7.8)	18.6 (11.4–25.7)	19.1 (14.0–24.1)	*p* < 0.001
Average amount of PA in minutes per day, mean (95% CI)	239.2 (210–268)	125.9 (96–155)	249.6 (224–275)	155.5 (119–192)	143.7 (122–165)	*p* < 0.001
Average amount of PA in MET/min/week, mean (95% CI)	8,232 (7,155–9,310)	4,009 (2,982–5,036)	8,064 (7,082–9,048)	4,633 (3,500–5,766)	4,701 (3,893–5,509)	*p* < 0.001

## Discussion

This study presents information on prevalence of physical inactivity and sedentary behavior in Armenia for the first time. In general, the level of physical inactivity in Armenia is slightly lower than the estimated average globally. A study of physical activity that included data from 168 countries estimated that 27.5% of the pooled sample were physically inactive (26). In Armenia, 21.6% of the population's did not meet the WHO recommendations of being physically active for at least 150 min per week. In terms of the Armenian population, this amounts to around 620 000 people whose health is at-risk due to physical inactivity.

The proportion of people who were physically inactive at the workplace was 47.2%, meaning that half the population have sedentary jobs. It is therefore essential that opportunities for PA be provided for people during working hours. This could include development of workplace health promotion policies which prioritize opportunities for PA throughout the day, supported by the development of infrastructure for PA and targeted interventions to encourage people to be active during working hours.

Another important finding is the low level of involvement in leisure-time PA. The rate of physical inactivity during leisure time in Armenia was 86.2%. These results are comparable to low- and middle-income countries and differ from high-income countries where leisure time PA is a major component of total PA undertaken by adults (27–29). Increasing the promotion and availability of sports and recreational facilities are a recommended priority area for health promotion in Armenia. More opportunities for PA during leisure time can be achieved through the development of infrastructure for recreational PA, investing in maintenance of existing parks, and encouraging sports clubs and facilities to develop programs and initiatives that promote physical activity and participation in sports for health.

The survey results also enabled the assessment and comparison of physical inactivity and sedentary behavior for different socio-demographic groups. Insufficient PA levels were more prevalent among older adults, Yerevan residents, as well as people who were unemployed, retired, or had a lower level of education. Total amount of PA was significantly lower among women, adults under 30 years old and over 60 years old, people with college or university degree, students, Yerevan and urban residents. Men spent more time sitting as did people under the age of 30 and over the age of 45, Yerevan residents, students, retirees and unemployed people.

Between men and women, no major differences were found in the proportion of people that did not meet WHO recommendations on PA. However, total amount of PA was significantly higher for men. This is consistent with results from other countries and the global trend (26, 29–31). Assessment of sedentary behavior time showed that despite their higher overall level of physical activity, men spent significantly more time sitting than women; 15.6% of men spent more than 8 h per day sitting which may have an adverse impact on health, independent of physical activity levels (7, 9, 11, 23, 24). Therefore, interventions to promote PA must target both men and women but different approaches may be required.

In developing countries, it has previously been observed that people in rural areas are more active in comparison with those living in urban areas (29, 32–34). This tendency was also found in Armenia where the highest level of physical inactivity and sedentary behaviors were in Yerevan, the capital city. Yerevan residents were less engaged in work- and transportation-related PA. Often in larger cities there are more opportunities for leisure time PA, however, 81.9% of the Yerevan population did not engage in any recreational PA. This indicates the need for additional measures to promote recreational PA and increasing accessibility of sports or recreational facilities should be taken into account in urban planning.

Another risk group with lower levels of PA is unemployed people. These results similar to other studies (35, 36). Unemployed people also spent more time sitting per day (over 4.5 h on average) and this segment of the population had the highest proportion of people who spent more than 8 h per day sitting (19.1%). This group should be targeted by interventions to increase PA and reduce SB which could include initiatives such as discounted user fees of recreational facilities to increase opportunities for participation in recreational PA.

Students in Armenia were significantly less active than other adults and spent more time sitting when compared with employed people and homemakers which is typical in other countries (28, 37, 38). Other studies showed that low PA levels of students has been associated with the university lifestyle, time spent studying for exams and academic pressure (39). Studying and preparing for exams require a lot of sitting time. Increasing PA levels of students may require strategies to increase transport- and recreation-related PA. There is potential to increase active transport to colleges and universities by prioritizing walking and cycling as part of urban planning and providing a range of recreational facilities to engage all students in leisure time physical activity.

The overall results of the study revealed a fairly high level of physical inactivity among the Armenian population. Health policymakers should prioritize elaboration of national policy documents on PA promotion. Development and implementation of programs for the promotion of PA and reducing of SB should be organized on national level and cover all at-risk groups. Leisure-time PA promotion can be a strategy to increase overall level of PA in the country.

Advantages of this study are a large and nationally-representative sample and the use of a standardized, validated questionnaire, and methodology. The use of GPAQ for monitoring current levels of PA and SB allowed the identification of those groups most at-risk due to high levels of physical inactivity and SB which provides important information for policy makers, researchers and health professionals when planning and implementing PA interventions. However, the GPAQ is a self-reported questionnaire and because of this the study has certain limitations. The understanding of vigorous- and moderate- intensity PA is subjective and responses may differ slightly depending on the participant or the interviewer conducting the survey. Also, in everyday life people generally do not track exactly how much time they have spent on a particular type of physical activity so responses depend on memory and a general estimate. Therefore, people may under- or over-report their physical activity and sedentary time. The use of objective methods to assess physical activity (such as accelerometers), even just in a sub-sample of the population to validate the responses to the questionnaires, could help to make the results more valid and reliable, and it is recommended that the inclusion of objective measurements be explored in future STEPS surveys and other studies of population physical activity levels. Another limitation is that the GPAQ allows to collect only amount of time spent sitting without specification of the activities performed during sedentary behavior (reading, watching TV, at the computer/tablet/mobile, etc.) or if person sits more at work or during leisure time. This information could help in developing more specific and effective interventions to reduce sedentariness. It should also be noted that although GPAQ has been validated extensively in various populations, it was not specifically validated for the Armenian population. This is also a limitation of the study and may be the subject of further research.

## Conclusions

This study provides baseline information on physical inactivity and sedentary behavior of the adult population in Armenia. This information could be highly useful for policy makers to develop a national action plan for physical activity and to guide the development of national PA guidelines to enable health professionals to promote PA. It could be used in urban planning, health promotion campaigns, and interventions targeted at specific groups in the population. Special attention should be paid to increase opportunities for recreational PA. The results of this WHO STEPS survey enable the identification of at-risk groups with high levels of physical inactivity and sedentary behavior, therefore providing information to develop more targeted, effective, and cost-effective PA interventions. This important baseline study provides the information needed to monitor and evaluate actions taken to increase PA and sedentary behavior and their impact on population PA levels as well as assessments of any resulting policy changes in Armenia.

## Data Availability Statement

The datasets generated for this study are available on request to the corresponding author.

## Ethics Statement

The studies involving human participants were reviewed and approved by the Ethics Committee of the National AIDS Prevention Center of Armenia and the WHO. The patients/participants provided their written informed consent to participate in this study.

## Author Contributions

AT, IR, and JB contributed conception and design of the study. DA supervised data collection. BM organized the dataset. AT and IR performed the statistical analysis. AT wrote the first draft of the manuscript with support from SW, IR, and JB. All authors contributed to manuscript revision, read and approved the submitted version.

## Conflict of Interest

The authors declare that the research was conducted in the absence of any commercial or financial relationships that could be construed as a potential conflict of interest.

## Abbreviations

GPAQ: Global Physical Activity Questionnaire
MET: metabolic equivalent of task
NCD: non-communicable disease
PA: physical activity
SB: sedentary behavior
WHO: World Health Organization.
